# Evaluation of Anatolian Water Buffalo Carcass Weights Based on a Slaughterhouse Data Collection

**DOI:** 10.3390/ani14050710

**Published:** 2024-02-24

**Authors:** Nursen Ozturk, Sevinc Arap, Omur Kocak, Lorenzo Serva, Kozet Avanus, Halil Ibrahim Kilic, Luisa Magrin, Halil Gunes

**Affiliations:** 1 Department of Animal Breeding and Husbandry, Faculty of Veterinary Medicine, Istanbul University-Cerrahpasa, 34500 Istanbul, Turkey; nursen.dogan@iuc.edu.tr (N.O.); okocak@iuc.edu.tr (O.K.); avanus@iuc.edu.tr (K.A.); halil.kilic@iuc.edu.tr (H.I.K.); gunes@iuc.edu.tr (H.G.); 2 Department of Animal Breeding and Husbandry, Institute of Graduate Studies, Istanbul University-Cerrahpasa, 34500 Istanbul, Turkey; sevincarap@gmail.com; 3 Department of Animal Medicine, Production and Health, University of Padova, 35122 Padova, Italy; luisa.magrin@unipd.it

**Keywords:** age, buffalo, sex, season, farm origin, fattening performance

## Abstract

**Simple Summary:**

Water buffalo husbandry is generally conducted for dairy purposes. However, buffalo meat is gaining growing importance due to its health benefits. To meet the increasing consumer demand, it is necessary to investigate the factors affecting the buffalo carcass weight. This study explored how the buffalo slaughter weight and age at slaughter were affected by various factors, including the sex, year of slaughter, season of slaughter, farm origin, and animal origin. Our results showed that the buffalo weight improved significantly by 46 kg throughout 2017–2021. Male buffalos and buffalos slaughtered in summer had higher carcass weights than their counterparts. Furthermore, we determined that the slaughter age and farm origin differed significantly between sexes. Since the buffalo carcass price in Turkey is based on quantity, our results can be implemented to achieve more efficient buffalo husbandry to increase farm incomes.

**Abstract:**

This study analyzed data collected on a slaughterhouse from 2017 to 2021, belonging to five hundred and twenty one Anatolian water buffalos from different farms located in Edirne, Istanbul, and Kirklareli. Specifically, it aimed to determine the factors affecting the carcass weights and slaughter ages of the Anatolian water buffalos. The results of the study showed that the slaughter age of the buffalos was a significant determinant of their carcass weights. Meanwhile, the sex, slaughter year, and slaughter season affected the carcass weight. Differences were observed for the slaughter age regarding the sex and farm origin. Since the pricing system in local markets is based on the buffalo carcass weight, the findings of this study could be essential for farmers when determining their fattening strategies.

## 1. Introduction

In Turkey, the main economic activity of a buffalo farm is based on milk production, where male buffalo calves are sent to slaughter without a specific fattening treatment and before attaining an optimal slaughter age [1]. Generally, the direct consumption of buffalo meat is minimal and it is less popular than cattle meat. For this reason, buffalo meat is mainly processed into sujuk (semi-dried fermented sausage) [2]. Between 2013 and 2021, there was a significant increase of 136.5% in buffalo meat production [3]. Ozturk et al. [4] found that dual-purpose farms in the Marmara region of Turkey have been practicing an intensive management system to diversify their economic activity by fattening buffalo calves. Moreover, the buffalo carcass weight improved by 28.8 kg/animal between 2001 and 2021 [5].

In the last decade, buffalo meat has increased in popularity globally because several nutritional and health advantages have been identified. Among the latter, a lower risk of cardiovascular diseases has been associated with buffalo meat consumption [6,7].

The rise of quality-assured buffalo meat brands like TenderBuff^®^ in Australia and Sapore di Campania in Italy has created a market opportunity but also requires producers to adhere to stringent quality requirements for their inclusion, related to the carcass weight and fat, protein, cholesterol, and iron content [8,9]. However, in many countries, due to a lack of quality-based pricing, farmers are driven to increase the animals’ carcass weights to obtain a significant economic advantage [10,11].

Several researchers have explored the many endogenous and exogenous factors affecting the carcass weights of buffalos, including the breed, sex, age, management practices, geographic locations of farms, and slaughter season [9,12,13]. Camcı and Erdem [14] and Ekiz et al. [15] revealed that sex significantly affected the carcass weight and that male buffalos achieved higher weights. In contrast, Turan et al. [16] reported that sex did not have a significant impact on buffalos’ carcass weights but the interaction between sex and age did. Specifically, young male and young female Anatolian water buffalos had higher carcass weights than their older counterparts. Moreover, they stated that the carcass weight also increased with age. Yılmaz et al. [17] reported that, under an intensive fattening strategy, male buffalos achieved a mean carcass weight of 325.4 kg, while females averaged 288.2 kg, highlighting a sex-based difference. Nikolaou et al. [18] emphasized the importance of providing additional feed during the finishing stage to achieve higher carcass weights, unlike an extensive grazing system on local pastures. Nikolaou et al. [13] pointed out that the geographical location of buffalo farms could significantly affect the carcass weights of animals due to different environmental conditions and farmer business profiles. Regional variations in consumers’ tastes, such as a higher demand for certain products, incentivize farmers to adapt their practices and become more professional. The slaughtering season may also influence the buffalos’ carcass weights. Omran et al. [12] reported that in hot seasons, buffalos reduced their feed intake due to heat stress, resulting in lower carcass weights. 

Buffalo husbandry in Turkey and almost worldwide is generally conducted by small–medium-scale family farms [2,4,9]. Although the buffalo meat sector has vast economic potential, these small farms fail to exploit this potential efficiently [9]. Therefore, new strategies that improve the buffalo carcass weight to satisfy the growing demand for meat production are needed.

This study aimed to provide a comprehensive descriptive analysis of the main characteristics of Anatolian water buffalos slaughtered at the governmental Edirne slaughterhouse between 2017 and 2021. Additionally, the study explored the relationships between the carcass weight, animal age, sex, year of slaughter, farm and animal origin, and slaughter season. The findings of this study will contribute towards greater knowledge of the intensive fattening systems of buffalos for different years and in various regions.

## 2. Materials and Methods

### 2.1. Dataset Description

Edirne (41°40′29.8740″ N and 26°35′0.5316″ E), Istanbul (41°8′39.228″ N and 28°27′36.7596″ E), and Kirklareli (41°44′2.2524″ N and 27°13′7.068″ E) are cities in the Marmara region in Turkish Thrace. In these provinces, lactating buffalos are generally sent to the pasture, except in winter, and they are supplemented with concentrate, grains, and roughage in addition to the pasture. A typical diet for female buffalos consists of concentrate sources supplemented by grains (mainly wheat, wheat shorts, wheat bran, and beet pulp) and roughage sources such as wheat straw, barley straw, alfalfa hay, and corn silage. Buffalo calves and males are reared indoors and fed total mixed rations (grains and roughage) [4].

Data collected at the slaughterhouse on the sex, age at the slaughter (months), and carcass weight (kg) of 521 Anatolian water buffalos from farms located in Edirne (*n* = 4), Istanbul (*n* = 4), and Kirklareli (*n* = 1) served as the basis for this study. The dataset was sourced from the governmental Edirne slaughterhouse.

In the slaughterhouse, the buffalos were kept off feed 24 h before slaughter but provided with free water in the paddocks. The carcass weight (in particular, the hot carcass weight) was determined as the slaughtered animal’s body weight after being skinned, bled, and eviscerated, and after removing the external genitalia, limbs, head, tail, kidneys, kidney fats, and scrotum or udder. The slaughter procedure was performed following the regulations on operational and inspection processes and the principles of red meat production facilities [19].

Data were gathered directly by experienced personnel from the slaughterhouse management software covering the years 2017 to 2021, without the need for animal handling.

### 2.2. Calculations and Statistical Analysis

The dataset’s descriptive analysis utilized medians and quartiles depicted through whisker box plots. Data normality was assessed using the Shapiro–Wilk test (values (W) ≥ 0.90 indicated a normal distribution). The differences in buffalo age at slaughter and carcass weight were estimated using ANOVA models, considering the breeding farm as a fixed effect. Moreover, data regarding the buffalo age at slaughter were analyzed with a mixed-effects ANOVA model that considered the fixed effects of the farm origin, animal origin, sex, year, and season, using the carcass weight as a covariate and breeding farm as a random effect. The buffalo carcass weights were analyzed with a similar mixed-effect ANOVA model that considered the fixed effects of the farm origin, animal origin, sex, year, and season, using the slaughter age as a covariate and the breeding farm as a random effect.

Post-hoc pairwise comparisons were tested between factor levels using Bonferroni correction. The assumptions of the linear model on the residuals were graphically tested. The minimum threshold of statistical significance was set at *p* < 0.05.

Statistical analyses of data were conducted by using XLSTAT (Addinsoft, release 2022.2.1, New York, NY, USA).

## 3. Results

The medians for the age at slaughter and carcass weight of buffalos were 25.0 months (21.8–32.3, first to third quartiles) and 248 kg (224–281, first to third quartiles), respectively (Supplementary Figure S1). The frequency of data collected for each category (breeding farm, sex, slaughter year, slaughter season, farm and animal origin) is reported in Supplementary Table S1. Descriptive outcomes for the carcass weights and age at slaughter of buffalos stratified by farm or sex are reported in Supplementary Figures S2 and S3, respectively.

**Figure 1:**
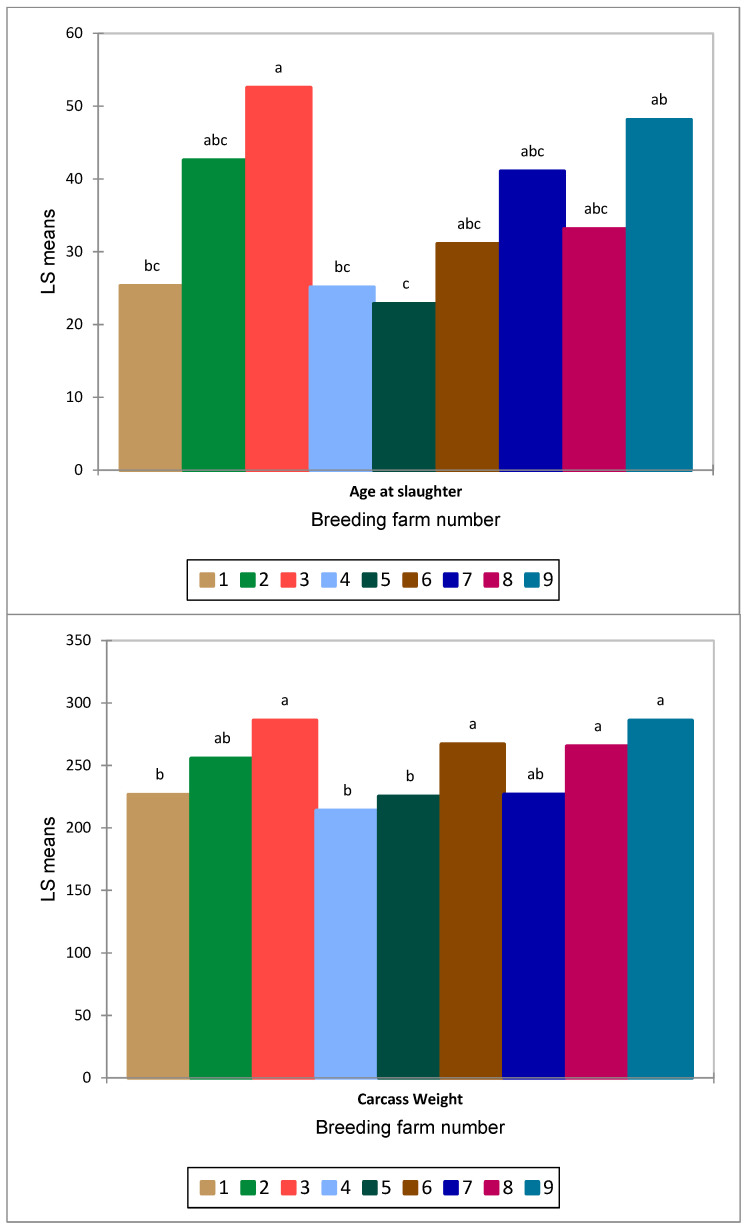
The effect of the breeding farm on the age at slaughter (months) and carcass weight (kg) of the Anatolian buffalo dataset (*n* = 521) was considered. Different lowercase letters indicate statistical differences in group means. Each farm is represented by a different colored column and coded from 1 to 9.

The breeding farm affected both the age (*p* < 0.0001) and carcass weight of the animals (*p* < 0.0001) (Figure 1), revealing that farms differed in the length of the breeding cycling period. However, based on the farm’s average, Equation (1) shows that the carcass weight decreased with age, with an R^2^ = 0.56 (RMSE = 7.60, *p* = 0.20).

Carcass weight (kg) = −37.148 + 0.291 × age (months)(1)

The outcomes of the mixed model revealed significant differences in the average carcass weight among sex, year, and season, while there was a tendency due to the farm origin. Moreover, the carcass weight did not differ with regard to the animal origin. The covariate of age at slaughter showed a coefficient of 0.254 (SE = 0.08, *p* = 0.002).

Furthermore, the latter mixed model showed significant differences in the average age at slaughter among sex and farm origin, while animal origin showed a tendential effect. However, the year and season of slaughter did not significantly affect the age at slaughter (Table 1). The covariate of carcass weight showed a coefficient of 0.078 (SE = 0.02, *p* = 0.001).

## 4. Discussion

This is the first study that has attempted to obtain insights into Anatolian water buffalo carcasses by analyzing the collection of slaughterhouse data corresponding to the 2017–2021 period. Although this study found that the carcass weights of buffalos regressed positively with increased age, in agreement with the results of other studies [13,20], it is necessary to underline that an optimum fattening time has multiple components. 

To rear a fattening animal profitably, a comprehensive understanding of the growth, fat accumulation, and feed intake dynamics is essential [21]. In this study, the objective was not to optimize the slaughter ages of the water buffalos but rather to ascertain the variations in buffalo fattening performance among farmers. We observed significant carcass weight differences for both sexes, the slaughter year, and the slaughter season. Male buffalos had higher carcass weights compared to female buffalos by 15 kg. The differences between the carcass weights were probably due to differences in the development of tissues and organs between males and females [10]. Male carcasses are mainly characterized by higher meat and lower fat content than female carcasses [22]. In line with our results, Ekiz et al. [15] and Camcı and Erdem [14] found a higher carcass weight for male buffalo carcasses. 

In the study area, there was a 46 kg/animal increase in the buffalo carcass weight between 2017 and 2021. Other studies examining trends in cattle carcass weights have reported improvements ranging from 9 to 11 kg over four years [13,23]. When comparing the four-year carcass weight improvements of buffalos and cattle, it is evident that there is a substantial enhancement in the buffalo carcass weight. This significant difference shows that the buffalo meat sector has the potential to adapt and respond to the increasing and changing consumer demands for animal-origin protein.

Significant differences in carcass weights were found among the slaughter seasons, with a difference of 23 kg between summer and winter. In the Marmara region, buffalos are reared indoors or tied in paddocks during winter and do not practice grazing. Therefore, farmers may struggle to supply their animals with highly nutritious feed (especially quality roughage). On the other hand, most of the buffalo farmers in the Marmara region send their animals to pasture during summer, while they continue to supply concentrate feed [4]. Considering the pasture availability in the area, we may speculate that buffalos slaughtered in the summer seasons show better carcass performance. During the study period, the warm climate in the Marmara region [24] did not negatively affect the animals’ carcass weights, which differs from the results of Om-ran et al. [12].

The farm region effect was not significant for the buffalo carcass weight. However, we determined a tendency. Buffalo carcasses in Istanbul had the highest weight (263 kg), followed by Edirne (235 kg) and Kirklareli (194 kg). A national project was initiated in 2011 with the purpose of improving Anatolian water buffalos’ milk and growth characteristics through genetic selection. As a consequence of the project, there was an improvement of 20.8 kg per animal in the live weights of buffalos in the 12th month of age in Istanbul between 2012 and 2017 [25]. The highest carcass weight of the buffalos from farms in Istanbul observed in this study could have resulted from the selection project, since Istanbul was the only province to adopt the selection project compared to the other two, Edirne and Kirklareli. Another contributing factor to the higher carcass weight may be the increased demand for buffalo products among consumers in Istanbul compared to Edirne and Kirklareli. Therefore, farmers in Istanbul may adopt more intensive strategies to enhance the carcass weights of buffalos [17,18].

However, this study had some limitations regarding the factors affecting the carcass weights of buffalos. For instance, other studies have shown that the feeding regime is a significant factor that affects the carcass weights of animals [26]. Moreover, the animals’ welfare condition before the slaughtering process, i.e., stress, anxiety, fear, and/or discomfort, is likely to impact their carcass weights [27,28]. In our study, the analysis did not include factors related to nutrition information or animals’ welfare conditions before slaughter. Therefore, additional research studies are essential for a complete understanding of all the factors affecting the carcass weights of buffalos. 

In this study, we observed that the slaughter age showed differences among the sex and farm origin. The average slaughter age for male and female buffalos was 27.0 and 41.6 months, respectively. Female buffalos are commonly kept for production and as replacements and are distinguished by a long and prolific lifespan of up to 24 years [29]. Meanwhile, male buffalos are mainly used for fattening and sent to slaughter at an optimal age, considering the economic and carcass weight values [18]. When comparing our results with the literature, the slaughter age was lower than the reported values for buffalos in Bulgaria [30] and Greece [18]. The average slaughter age of female buffalos observed in this study (41.6 months) was found to be similar to the slaughter age of female cattle (44.1 months) [13]. One potential explanation for this trend could be the buffalo farmers’ adoption of more intensive strategies to enhance milk production, as evidenced by recent studies [4,31]. This may involve the practice of slaughtering females once their milk production starts to decline, similar to methods employed in intensive dairy cattle farming.

In the present study, farmers exhibited varying performance in their decisions to send their animals to slaughter. For instance, the slaughter age for animals from farms in Istanbul was the highest (43.7 months) as compared to those from farms in Edirne (33.4 months) and Kirklareli (25.6 months). As indicated by Salari et al. [10], farmers delay the slaughter age to obtain higher carcass weights, because the carcass weights of older animals are higher than those of young animals [32]. Hence, it is reasonable to infer that buffalo farmers in Istanbul demonstrate a higher level of professionalism in their livestock management practices and exhibit a business-oriented profile that prioritizes their economic performance. Although there was a tendency for a significant effect of the animal origin on the slaughter age, all the carcasses belonged to the Anatolian water buffalo; therefore, we do not expect any existing impact of this variety on the growth performance. 

## 5. Conclusions

In Turkey, buffalo farmers are incentivized to enhance the carcass weight due to the significantly higher prices offered for heavier carcasses. Therefore, understanding the factors affecting buffalo carcass weights is crucial. In our study, the weight of the buffalo carcasses increased with age, and it was significantly different according to the sex, year, and slaughter season. Furthermore, farmers exhibited varying performance concerning the slaughter ages of their animals. With the notable improvement in carcass weight observed since 2017, it is evident that the buffalo meat sector is undergoing development. Future studies are needed to determine the optimum slaughter age of the buffalo considering the growth rate, fat deposition characteristics, and feed intake dynamics for more profitable production.

## Figures and Tables

**Table 1:** The effects of sex, slaughter year and season, animal and farm origin, and age at slaughter (as a covariate) on the carcass weights of buffalos. The effects of sex, slaughter year and season, animal and farm origin, and carcass weight (as a covariate) on the age at slaughter of buffalos. The breeding farm was used as a random effect for both mixed ANOVA models.

**Carcass Weight (kg)**																			
Sex				Slaughter Year				Slaughter Season				Farm Origin				Animal Origin			
	value	*p*	SEM		value	*p*	SEM		value	*p*	SEM		value	*p*	SEM		value	*p*	SEM
Male	238	0.001	8.76	2021	249 a	<0.0001	9.42	Winter	219 c	<0.0001	9.13	Istanbul	263	0.081	10.3	Istanbul	238	0.826	12.8
Female	223			2020	241 a			Spring	225 bc			Edirne	235			Edirne	235		
				2019	232 a			Summer	242 a			Kirklareli	194			Tekirdag	230		
				2018	228 a			Autumn	238 ab							Kirklareli	219		
				2017	203 b														
**Age at slaughter (months)**																			
Sex				Slaughter Year				Slaughter Season				Farm Origin				Animal Origin			
	value	*p*	SEM		value	*p*	SEM	value		*p*	SEM		value	*p*	SEM		value	*p*	SEM
Male	27.0 b	<0.0001	4.54	2021	33.9	0.175	4.91	Winter	33.1	0.299	4.75	Istanbul	43.7	0.045	5.39	Istanbul	35.4	0.081	6.67
Female	41.6 a			2020	34.4			Spring	36.5			Edirne	33.4			Edirne	28.7		
				2019	38.5			Summer	35.8			Kirklareli	25.6			Tekirdag	44.1		
				2018	31.7			Autumn	31.7							Kirklareli	28.9		
				2017	32.9														

SEM = Standard Error of the Mean. Different lowercase letters indicate significant differences in the means at *p* < 0.05.

## Data Availability

The data presented in this study are available upon reasonable request from the corresponding authors.

## Supplementary Materials

The following supporting information can be downloaded at: https://www.mdpi.com/article/10.3390/ani14050710/s1, Figure S1: A box plot showing the age (months) and carcass weight (kg) of the Anatolian buffalo dataset. Figure S2: A box plot showing the nine farms in the Anatolian buffalo dataset; Figure S3: A box plot showing the females and males in the Anatolian buffalo dataset; Table S1: The frequencies for the breeding farm, sex, slaughter year and season, farm origin, and animal origin in the Anatolian buffalo dataset.
