# Supplementary material for: Evaluation of Anatolian Water Buffalo Carcass Weights Based on a Slaughterhouse Data Collection
Source: Animals (Basel). 2024 Feb 24;14(5):710. doi: 10.3390/ani14050710 (PMC10931152; doi:10.3390/ani14050710)
Supplement: Supplementary file 1 [file animals-14-00710-s001.zip › animals-2842305-supplementary.pdf]

Supplementary Figures

Supply. Figure S1: The median for age at slaughter and carcass weight of buffalos.

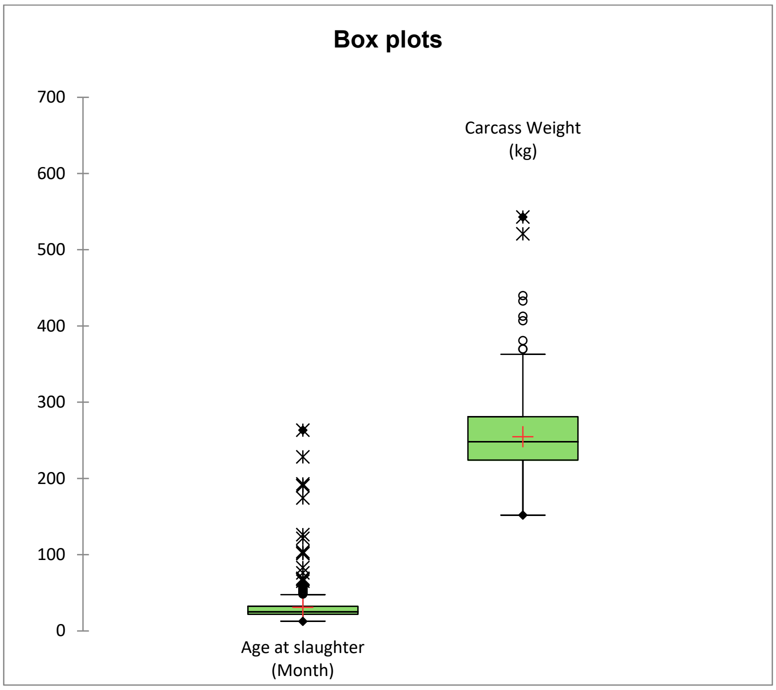

The box plot for age (months) and carcass weight (kg) of the Anatolian buffalo dataset (n = 521). The red cross represents the mean, and horizontal dark solid lines represent (from bottom to up) the lower limit (expressed as first quartile - 1.5 × (third quartile - first quartile)), the first, second and third quartiles, and upper limit (expressed as third quartile + 1.5 × (third quartile - first quartile)). Symbols exceeding lower to upper intervals are possible outliers. Solid black circles represent the minimum and maximum values.

Supply. Figure S2: The descriptives for age at slaughter and carcass weight of the buffalos are depicted for the farm classification.

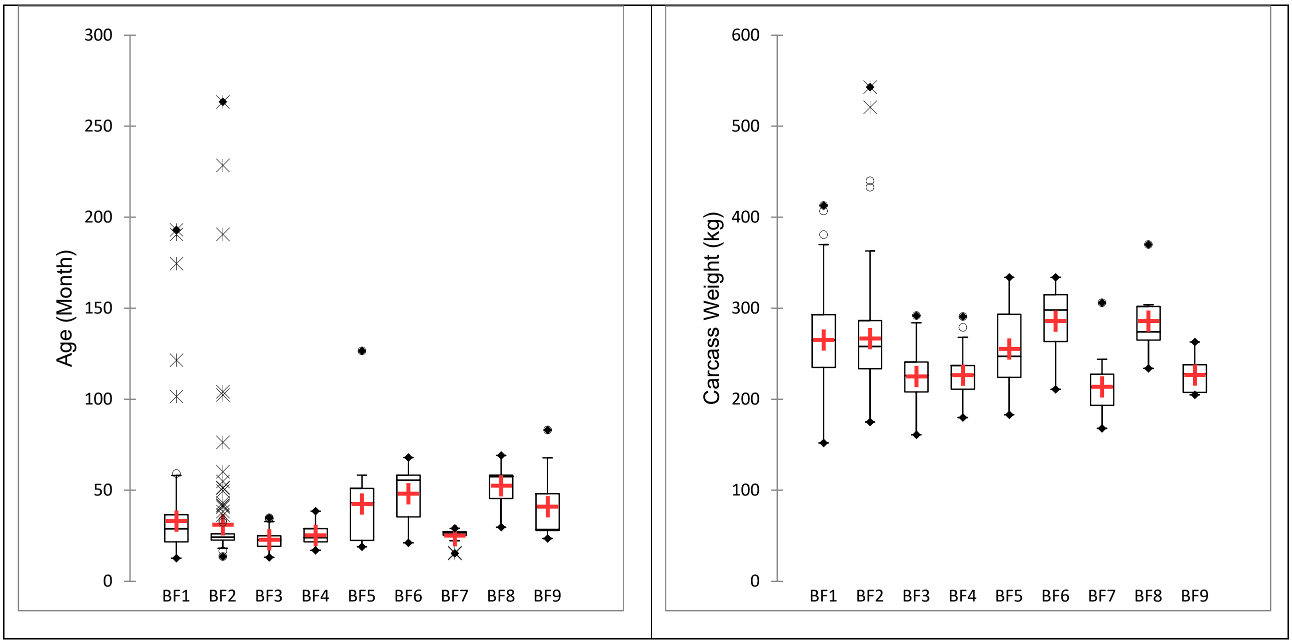

The box plot for the nine farms in the Anatolian buffalo dataset (n = 521). The red cross represents the mean, and horizontal dark solid lines represent (from bottom to up) the lower limit (expressed as first quartile - 1.5 × (third quartile - first quartile)), the first, second and third quartiles, and upper limit (expressed as third quartile + 1.5 × (third quartile - first quartile)). Symbols exceeding lower to upper intervals are possible outliers. Solid black circles represent the minimum and maximum values. BF = Breeding farm number.

Supply. Figure S3: The descriptives for age at slaughter and carcass weight of buffalos are depicted for the sex classification.

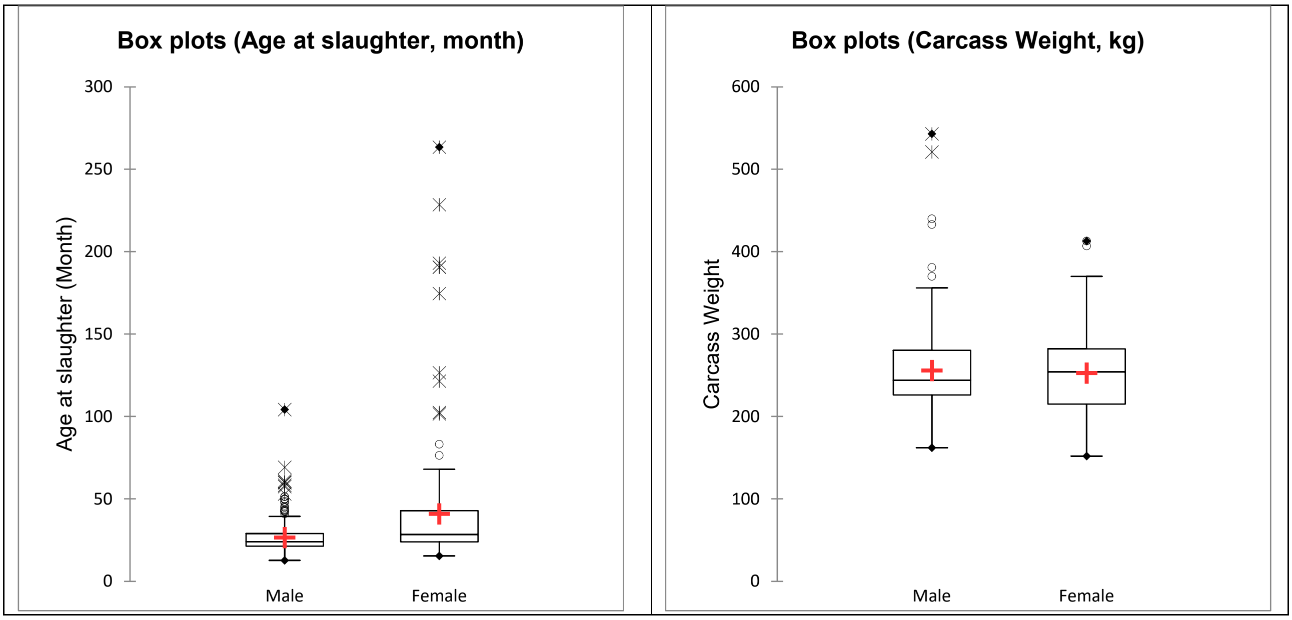

The box plot for females and males in the Anatolian buffalo dataset (n = 521). The red cross represents the mean, and horizontal dark solid lines represent (from bottom to up) the lower limit (expressed as first quartile - 1.5 × (third quartile - first quartile)), the first, second and third quartiles, and upper limit (expressed as third quartile + 1.5 × (third quartile - first quartile)). Symbols exceeding lower to upper intervals are possible outliers. Solid black circles represent the minimum and maximum values.

Supplementary Tables

Supply. Table S1: The frequency for farm code, sex, slaughter year and season, farm origin, and animal origin in the buffalo dataset (n = 521).

Variable	category	Frequency per category
Breeding farm	1	185
	2	147
	3	83
	4	43
	5	18
	6	15
	7	14
	8	9
	9	7
Sex	Female	169
	Male	352
Slaughter Year	2017	61
	2019	143
	2018	110
	2020	114
	2021	93
Slaughter Season	Winter	121
	Spring	118
	Summer	161
	Autumn	121
Farm Origin	Edirne	458
	Istanbul	49
	Kirklareli	14
Animal origin	Edirne	240
	Istanbul	270
	Kirklareli	3
	Tekirdag	8
